# Efficacy and Safety of Diaphragmatic Breathing Exercises for Gastroesophageal Reflux Disease: A Systematic Review and Meta-Analysis

**DOI:** 10.3390/jcm15093406

**Published:** 2026-04-29

**Authors:** Omar Abureesh, Faris Qaqish, Mohammad Abu-Shaban, Chloe Lahoud, Toni Habib, Joelle Sleiman, Elie Moussa, Youssef El Douaihy, Jean Chalhoub, Sherif Andrawes

**Affiliations:** 1 Department of Internal Medicine, Staten Island University Hospital, Staten Island, NY 10305, USA; fqaqish@northwell.edu (F.Q.);; 2 Department of Internal Medicine, Southeast Georgia Health System, Brunswick, GA 31520, USA; 3 Division of Gastroenterology and Hepatology, Department of Internal Medicine, Staten Island University Hospital, Staten Island, NY 10305, USAjchalhoub@northwell.edu (J.C.);

**Keywords:** diaphragmatic breathing, gastroesophageal reflux disease, GERD, lifestyle intervention, proton pump inhibitors, quality of life, systematic review, meta-analysis

## Abstract

**Background:** Gastroesophageal reflux disease (GERD) is a highly prevalent gastrointestinal disorder worldwide. Management strategies include lifestyle modification, pharmacologic therapy, and surgical interventions. Diaphragmatic breathing exercises have been proposed as a non-pharmacological treatment aimed at improving lower esophageal sphincter function and reducing reflux episodes. **Methods:** A systematic search of PubMed/MEDLINE, Scopus, ScienceDirect, Google Scholar, and ClinicalTrials.gov was conducted from database inception to 10 March 2026 to identify randomized controlled trials evaluating diaphragmatic breathing in patients with GERD. Two reviewers independently screened studies, extracted data, and assessed risk of bias using the Cochrane Risk of Bias 2.0 tool. Random-effects meta-analyses were performed to estimate pooled mean differences for symptom scores and quality-of-life outcomes. **Results:** Ten randomized controlled trials including 476 patients were analyzed (mean age: 39.9 ± 11.3 years). Diaphragmatic breathing interventions were performed in 229 participants, with an average duration of 20.36 min per session over approximately 5.1 weeks. Meta-analysis demonstrated a modest improvement in GERD symptom scores favoring diaphragmatic breathing (SMD −0.74; 95% CI −1.36 to −0.12; *p* = 0.019), with substantial heterogeneity (*I*^2^ = 79.7%). Subgroup analyses comparing breathing with medication and sham breathing controls produced similar trends. Quality-of-life outcomes did not demonstrate statistically significant improvement (MD −2.35; 95% CI −6.35 to 1.65; *p* = 0.25) and showed considerable heterogeneity (*I*^2^ = 85.3%). Risk-of-bias assessment revealed “some concerns” in several studies, primarily related to randomization procedures and outcome reporting. **Conclusions:** Although pooled results demonstrated a statistically significant reduction in GERD symptom scores favoring diaphragmatic breathing, this finding must be interpreted with considerable caution given the substantial heterogeneity observed. The current evidence remains limited by methodological heterogeneity, and inconsistent outcome assessment is insufficient to support definitive clinical recommendations, and the observed benefit may not be generalizable across patient populations or clinical settings. Larger standardized randomized trials are required to determine the clinical role of diaphragmatic breathing in GERD management.

## 1. Introduction

Gastroesophageal reflux disease (GERD) is one of the most prevalent gastrointestinal disorders worldwide. It is estimated that 10–15% of Western populations are affected by this condition, with an estimated incidence rate of 4.5 cases per 1000 patients per year in the United Kingdom [1,2]. The Montreal Consensus defines GERD as “a condition which develops when the reflux of stomach contents causes troublesome symptoms and/or complications” [3]. This definition allows for a patient-centered, technology-independent, inclusive, and diverse detection of various GERD types [3]. Clinically, GERD is classified into two main categories: esophageal syndromes and extra-esophageal syndromes [2].

Esophageal syndromes are often associated with typical reflux syndrome, reflux chest pain syndrome, reflux esophagitis, Barrett’s esophagus, and adenocarcinoma. These syndromes predominantly manifest as troublesome heartburn (a burning sensation in the chest) and/or regurgitation (the perception of gastric content flowing into the mouth). Severe episodes of chest pain can also occur, mimicking ischemic cardiac pain even in the absence of typical heartburn or regurgitation [3,4,5,6]. Conversely, extra-esophageal syndromes are associated with chronic cough, chronic laryngitis, asthma, and dental erosion. While GERD is recognized as a potential cofactor for these conditions, it is rarely the sole cause [7,8,9,10].

**Figure 1:**
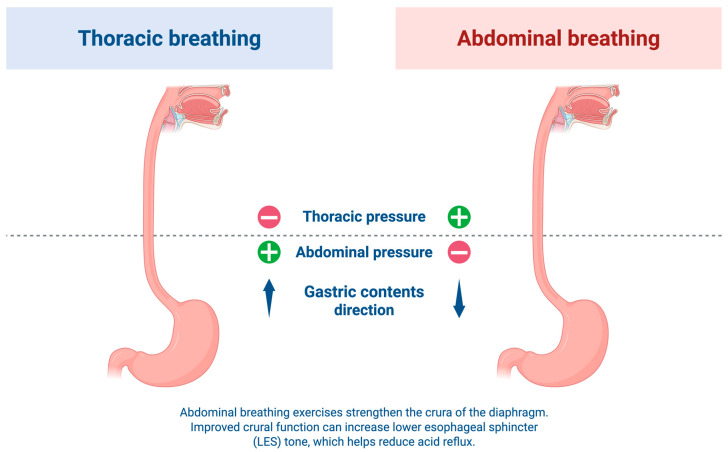
Impact of breathing patterns on thoracoabdominal pressure gradients and gastroesophageal reflux dynamics. These schematic contrasts the physiological effects of thoracic versus abdominal breathing on the gastroesophageal junction. (**Left**) During shallow thoracic breathing, the thoracoabdominal pressure gradient (characterized by negative intrathoracic pressure and positive intra-abdominal pressure) favors the proximal migration of gastric contents, facilitating reflux. (**Right**) Conversely, abdominal (diaphragmatic) breathing alters this pressure dynamic, promoting a downward vector that resists reflux. Furthermore, consistent abdominal breathing exercises actively strengthen the crural fibers of the diaphragm. This enhanced crural function mechanically augments the basal tone of the lower esophageal sphincter (LES), creating a more competent anti-reflux barrier and mitigating gastroesophageal reflux episodes.

Several treatment options for GERD are currently available. The 2022 American College of Gastroenterology (ACG) guidelines categorize management options into lifestyle modifications, medical therapy, and surgical or endoscopic interventions [11]. Among medical treatments, proton pump inhibitors (PPIs) are the most commonly prescribed and are considered highly effective, particularly for non-erosive reflux disease (NERD) and erosive esophagitis (EE) [12]. However, long-term PPI use is associated with adverse effects, including decreased magnesium absorption and a higher risk of osteoporosis-related fractures, as well as potential intestinal infections and poor vitamin and mineral absorption [13,14]. Consequently, diaphragmatic breathing has been suggested as a non-pharmacological alternative or adjunct for GERD patients. Because the crural diaphragm functions as the external lower esophageal sphincter, targeted breathing exercises are thought to prevent reflux by strengthening this anatomical barrier [15,16]. Figure 1 illustrates the impact of breathing patterns on thoracoabdominal pressure gradients and gastroesophageal reflux dynamics. This systematic review and meta-analysis investigates the efficacy of diaphragmatic (abdominal) breathing for GERD treatment compared to standard therapies.

## 2. Methods

### 2.1. Study Design

The PRISMA checklist and Cochrane criteria were followed in conducting this systematic review [17,18]. A detailed protocol was pre-registered at PROSPERO registry under the number (CRD420261299564). Ready PRISMA checklist and PROSPERO protocol report can be found in the Supplementary Materials [19,20].

### 2.2. Inclusion Criteria

The primary outcome of interest was the evaluation of GERD symptom improvement. Studies that compared the effectiveness of diaphragmatic (abdominal) breathing with conventional PPI therapy or sham breathing techniques were included in this analysis. To be included, studies had to meet the following predefined criteria:

1. Randomized controlled trials (RCTs) evaluating diaphragmatic breathing interventions in patients diagnosed with GERD.
2. Clinical studies that simultaneously examined diaphragmatic breathing against conventional PPI therapy or sham breathing techniques.
3. Articles published in English.

### 2.3. Search Strategy and Study Selection

A comprehensive systematic search of the PubMed/MEDLINE, Google Scholar, and ScienceDirect databases was conducted from database inception until 10 March 2026. “Abdominal breathing” [Mesh] and “GERD” [Mesh] were used as primary search terms. For Google Scholar, Web of Science, and ClinicalTrials.gov, we utilized the primary keywords: “abdominal breathing exercise AND GERD” without filters. For PubMed and Scopus, the following search string was applied ((“abdominal breathing”[MeSH Terms] OR “diaphragmatic breathing”[MeSH Terms] OR “diaphragmatic breathing”[tiab] OR “abdominal breathing”[tiab]) AND (“gastroesophageal reflux”[MeSH Terms] OR “GERD”[tiab] OR “erosive esophagitis”[tiab])). We also manually searched the reference lists of recognized systematic reviews published in this area for potentially relevant studies. Following the searches, references were exported to an EndNote X9 file, and duplicates were eliminated. Study screening and data extraction were performed by two independent reviewers, with any disagreements resolved by a third reviewer.

### 2.4. Risk-of-Bias Assessment

The methodological quality of the included studies was assessed using the Cochrane Risk of Bias 2.0 (RoB 2) tool [21]. Two sets of two reviewers completed this assessment independently, and any discrepancies were resolved through group discussion.

### 2.5. Data Acquisition

Two randomly selected authors extracted each paper independently to avoid bias, and a third reviewer settled any disputes. The collected data included the primary outcome (GERD scores), intervention descriptions, participant age and gender, country of origin, and study design. All procedures adhered to the methods recommended by the Cochrane Handbook for Systematic Reviews of Interventions [22].

### 2.6. Data Analysis

A random-effects model was used in a planned meta-analysis. Mean differences and 95% confidence intervals are displayed. Both quantitative and qualitative methodologies were used to analyze the collected data. Tables were used to narratively report patient and study characteristics. All continuous outcome meta-analyses (SMD and MD) were conducted using the DerSimonian–Laird random-effects model, consistent with the high *I*^2^ values observed (*I*^2^ = 79.7% for symptom scores; *I*^2^ = 85.3% for QoL). A fixed-effect model meta-analysis with 95% confidence intervals and proportion as the effect measure was used to examine patient survival. Standardized mean difference (SMD) or mean difference (MD) meta-analyses were used to examine continuous variables. The threshold for statistical significance was established at *p* < 0.05. *I*^2^ and Cochran’s Q statistics were used to measure heterogeneity, and they were interpreted in accordance with the Cochrane Handbook for Systematic Reviews. We used a random-effects model when significant heterogeneity (*I*^2^ > 50%) was found. We used the leave-one-out technique to find research that caused heterogeneity and identification of the study(ies) whose removal most substantially reduced *I*^2^, the resulting pooled SMD after removal, and an interpretation of whether the primary finding remained robust. The relevant study was removed from the synthesis if considerable heterogeneity remained after converting to a random-effects model. R software (version 4.12 of the meta package; R Foundation for Statistical Computing; Vienna, Austria) was used to perform statistical analyses. Funnel plot and Egger’s test were not performed because fewer than ten studies were included in each pooled analysis.

### 2.7. Publication Bias

According to Egger et al., evaluation of publication bias for less than ten pooled studies is unreliable. Given that the included studies were predominantly small, single-center, and randomized, the possibility of publication bias cannot be excluded. Studies reporting negative effects of diaphragmatic breathing may remain unpublished, which could lead to an overestimation of the pooled treatment effect. As a result, we were unable to employ Egger’s test for funnel plot asymmetry to evaluate publication bias in the current study [23,24].

## 3. Results

### 3.1. Search Results

**Figure 2:**
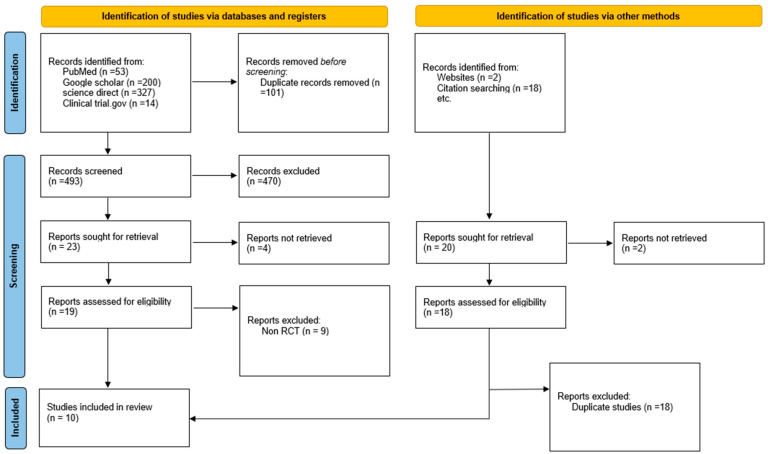
PRISMA 2020 flow diagram illustrating the systematic search and study inclusion process.

The initial systematic search yielded a total of 614 records, comprising 594 from electronic databases and 20 from citation searching and websites. After removing 101 duplicate records from the database searches, the remaining 493 studies were screened by title and abstract, which led to the exclusion of 470 records. Subsequently, 37 full-text reports (19 from databases and 18 from other sources) were assessed for eligibility. Following the exclusion of 27 reports (9 non-RCTs and 18 duplicate studies), a final total of 10 randomized controlled trials met all predefined criteria and were included in the review (Figure 2).

### 3.2. Characteristics of Included Studies and Patients

Included studies were all randomized clinical trials (RCTs). One study, Sun et al., was conducted as a pilot RCT with an open-label protocol [25]. Studies collectively described 476 patients with a mean age of 39.9 ± 11.3 years and a balanced sex distribution (53.13% male). Criteria for GERD diagnosis varied among the trials, encompassing different disease subtypes such as classic GERD, post-COVID-19 GERD, and non-cardiac chest pain (NCCP) GERD. The general characteristics of the included studies are summarized in Table 1.

On the other hand, 227 patients were managed using alternative (comparator) intervention. This group of patients had a mean age of 42.4 ± 11.6 years, and alternative interventions varied between standard medical therapies like PPIs, or Sham interventions. Table 2 and Table 3 describe the characteristics of interventions in the intervention and comparator groups, respectively. The comparator group consisted of 227 patients with a mean age of 42.4 ± 11.6 years. Control interventions varied, ranging from standard medical therapies (e.g., PPIs) to sham breathing exercises. Table 2 and Table 3 detail the intervention characteristics for the experimental and comparator groups, respectively.

### 3.3. Efficacy

A random-effects meta-analysis was conducted to evaluate the differences in efficacy between diaphragmatic breathing and standard therapy. Subgroup analysis was employed to stratify studies based on their control groups into breathing (sham/alternative) and medication controls. Overall, a statistically significant reduction in GERD symptom scores was found favoring diaphragmatic breathing, with a pooled standardized mean difference (SMD) of −0.74 (95% CI: −1.36 to −0.12, *p* = 0.019). However, substantial statistical heterogeneity was observed (*I*^2^ = 79.7%, *p* < 0.01). Neither subgroup independently reached statistical significance.

Within the breathing control subgroup, gastroesophageal reflux disease questionnaire (GERDQ) scores showed a trend favoring the abdominal breathing regimen (SMD = −0.74; 95% CI: −2.06 to 0.57), though this did not reach statistical significance, and heterogeneity remained high (*I*^2^ = 85.9%, *p* < 0.01). Similarly, in the medication control subgroup, symptom scores favored the breathing regimen (SMD = −0.73; 95% CI: −1.58 to 0.12) but were not statistically significant independently, alongside high heterogeneity (*I*^2^ = 83.0%, *p* < 0.01).

**Figure 3:**
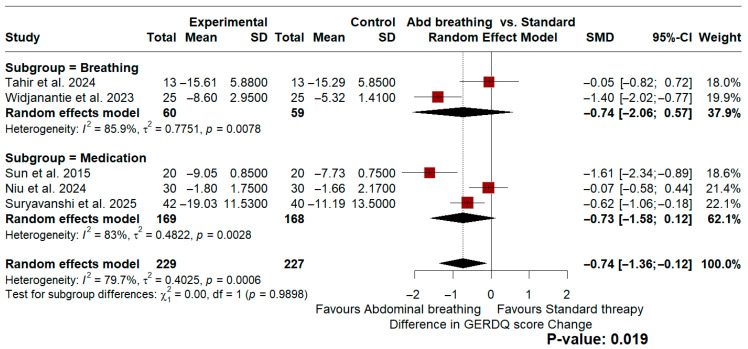
Forest plot presenting the random-effect model meta-analysis comparing the efficacy of abdominal breathing to standard therapy [25,26,28,29,31].

**Figure 4:**
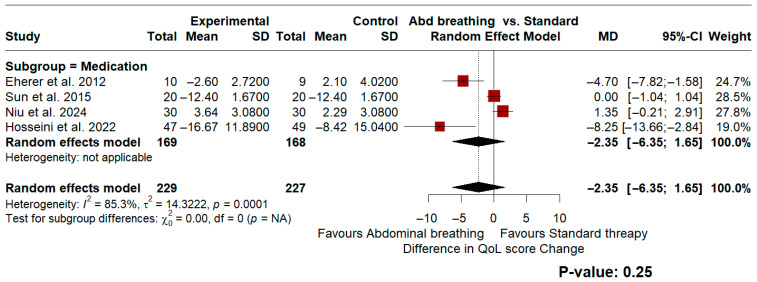
Forest plot presenting the random-effect model meta-analysis comparing the efficacy of abdominal breathing to standard therapy in QoL improvement [25,31,32,33].

A secondary analysis was conducted to assess improvements in quality-of-life (QoL) scores. For QoL measurements, all included studies utilized a medication control regimen. A pooled mean difference (MD) of −2.35 (95% CI: −6.35 to 1.65, *p* = 0.25) was observed. This indicated no statistically significant difference between the interventions for QoL improvement, accompanied by significant data heterogeneity (*I*^2^ = 85.3%, *p* < 0.01). Figure 3 and Figure 4 illustrate the forest plots for these described analyses.

### 3.4. Risk-of-Bias Assessment Results

**Figure 5:**
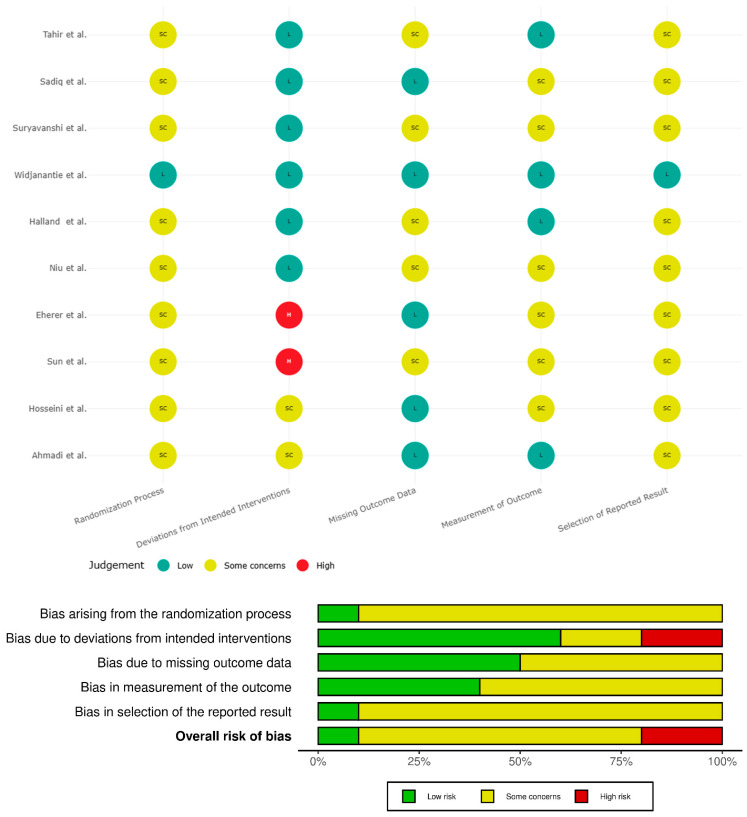
Traffic-light plot illustrating risk-of-bias assessment for included studies [25,26,27,28,29,30,31,32,33,34].

Risk of bias in included studies was assessed using Cochrane risk of bias 2.0 (RoB 2) assessment tool. Most included studies had some concerns in the domains of randomization, outcome measurement and reporting. Due to deviation from intended intervention, two studies (Sun et al. [25] and Eherer et al. [32]) were of high risk of bias. Figure 5 illustrates induvial and overall risk-of-bias assessment results.

## 4. Discussion

The present review investigates the efficacy of diaphragmatic (abdominal) breathing exercises as a management strategy for GERD. Our pooled cohort included 448 patients with a mean age of 39.9 ± 11.3 years. This is lower than the average age of GERD diagnosis reported in a previous epidemiological study by Richter and Rubenstein [35]. Their study reported an overall mean age at diagnosis of 50 ± 1.32 years, though with an *I*^2^ value of 91.5%, indicating high heterogeneity and suggesting these estimates should be interpreted with caution. In our cohort, most studies utilized an upper age limit of 60 years for inclusion, which may have artificially lowered the mean age of our patient population. Furthermore, the criteria for GERD diagnosis varied among the included trials, reflecting the broad range of etiologies associated with the disease. In the literature, the predominant mechanism responsible for most reflux episodes is transient lower esophageal sphincter (LES) relaxation. Other physiological abnormalities contributing to GERD include a hypotensive LES, swallow-associated relaxations, and strain-induced reflux [36]. In our review, the broad etiological definitions of GERD across the trials likely contributed to the substantial heterogeneity of our findings, which will be further explored below.

Across the included studies, the diaphragmatic breathing regimens averaged 20.36 min per session and were implemented over a mean duration of 5.11 weeks. This timeframe is clinically relevant, as it aligns with guideline-recommended PPI regimens; empirical PPI therapy is typically prescribed for 8 weeks, though a 4-week course is often sufficient for non-erosive reflux disease (NERD) [37,38]. Furthermore, breathing sessions were supervised in nearly all the trials. Because these physical interventions are highly technique-dependent, direct patient supervision was deemed necessary to ensure the correct execution of the exercises.

Beyond supervision, exercise frequency appears to be a critical determinant of success. Unlike other skeletal muscles, the diaphragm has a unique maintenance mechanism and a significantly faster recovery time [39], meaning infrequent training may fail to sustain therapeutic benefits. This observation is consistent with the trends identified in our meta-analysis (Figure 3); for instance, the study by Tahir et al., which employed the lowest frequency among included articles (two sessions per week), demonstrated a negligible effect (SMD −0.05) [26]. In contrast, studies implementing daily or multiple daily protocols demonstrated significantly greater magnitudes of symptomatic attenuation. This suggests that high-frequency stimulation is likely required to effectively reinforce the mechanical anti-reflux function of the crural diaphragm.

To avoid mixed-control comparisons, subgroup analyses were conducted to evaluate the reduction in GERDQ scores between the intervention and respective control groups. In trials utilizing an alternative or sham breathing technique as a comparator, the results demonstrated a standardized mean difference (SMD) of −0.74 (95% CI: −2.06 to 0.57) favoring the diaphragmatic breathing regimen, with significant heterogeneity observed (*I*^2^ = 85.9%, *p* < 0.01 for heterogeneity). Similarly, in the medication-controlled group, the SMD was −0.73 (95% CI: −1.58 to 0.12) favoring breathing exercises, again accompanied by high heterogeneity (*I*^2^ = 83.0%, *p* < 0.01). Neither subgroup independently reached statistical significance, and the overall pooled significance should therefore be interpreted cautiously, as it may be driven by aggregation across heterogeneous comparator arms rather than a robust treatment effect. Furthermore, the analysis of quality-of-life (QoL) improvement revealed a mean difference of −2.35 (95% CI: −6.35 to 1.65; *p* = 0.25), which was not statistically significant and also exhibited substantial heterogeneity (*I*^2^ = 85.3%, *p* < 0.01). Importantly, these observed differences may lack true clinical significance; because the GERDQ score and QoL assessments rely heavily on subjective patient reporting, they cannot be solely relied upon to assess actual clinical improvement [40].

Non-pharmacological interventions are often perceived favorably by patients compared to chronic medication use. In the context of GERD management, lifestyle modifications have shown potentially beneficial under limited and heterogeneous evidence results, which can be attributed to both the mechanical disruption of the pathophysiological mechanisms underlying the disease and the psychological benefits of non-medical management [15,41]. Ideally, clinical efficacy should be measured using objective, quantitative tools, such as lower esophageal sphincter (LES) pressure (mmHg), diaphragmatic thickening fraction (%), or maximum inspiratory pressure (cmH_2_O). While some of the reviewed studies assessed these parameters, they were not utilized by a sufficient number of trials to permit pooled meta-analysis. Consequently, the reliance on unstandardized GERD diagnostic criteria and subjective symptom assessments across the literature results in significant data heterogeneity, rendering the observed symptomatic improvements difficult to generalize clinically. A previous systematic review by Zdrhova et al. investigating breathing exercises for GERD reached a similar conclusion [16]. They suggested that breathing exercises can potentially improve esophageal motility and reduce reflux episodes by increasing LES pressure and enhancing diaphragmatic function. Nevertheless, consistent with the present study, their review highlighted that substantial heterogeneity in intervention protocols and outcome measures severely limits the ability to draw definitive clinical conclusions. However, beyond pharmacological therapy, several non-breathing lifestyle interventions have suggested a possible benefit that warrants further investigation in GERD management, including weight loss, elevation of the head of the bed, dietary modifications, and avoidance of recumbent positioning postprandially. Compared to these interventions, diaphragmatic breathing exercises are notable for their mechanism specificity, targeting the crural diaphragm directly rather than reducing reflux triggers. However, unlike dietary and positional interventions which are supported by higher-quality evidence and are already embedded in clinical guidelines, diaphragmatic breathing remains investigational, and its additive benefit over standard lifestyle modification has not yet been assessed in a head-to-head trial [11].

The findings of this study must be interpreted in light of several limitations. First, the criteria for GERD diagnosis varied widely among the included studies, resulting in significant data heterogeneity that could not be fully resolved through subgroup categorization or statistical adjustments. Second, the assessment of GERD improvement lacked a standardized approach. The reliance on surveys and non-quantitative tools introduces considerable subjectivity, further contributing to the inherent data heterogeneity observed. Third, because diaphragmatic breathing is not yet a guideline-standardized therapeutic maneuver, the included trials utilized highly variable intervention protocols (e.g., differing session lengths, frequencies, and durations), complicating data interpretation. Finally, the comparator arms were equally unstandardized. The control groups varied significantly, ranging from sham breathing exercises to various medications within the PPI class, which possess differing efficacy and safety profiles, thereby limiting direct comparability across studies. The findings of the present study remain reliant on subjective patient-reported symptom scores, and suffer from the lack of objective physiological endpoints (e.g., LES pressure, pH monitoring) since none were available for pooled analysis. Therefore, the clinical significance of the observed SMD of −0.74 remains uncertain and cannot be translated directly into a practice recommendation.

To address these gaps, future research must prioritize methodological standardization. Subsequent clinical trials should employ uniform criteria for GERD diagnosis, such as those established by the American College of Gastroenterology (ACG) guidelines, alongside standardized breathing protocols and objective, quantitative outcome measures. Furthermore, rigorous, high-quality randomized controlled trials with larger sample sizes are required to enhance statistical power and permit a more definitive, comprehensive evaluation of this potential management modality.

## 5. Conclusions

Although pooled results demonstrated a statistically significant reduction in GERD symptom scores favoring diaphragmatic breathing (SMD −0.74; 95% CI −1.36 to −0.12; *p* = 0.019), this finding must be interpreted with considerable caution given the substantial heterogeneity observed (*I*^2^ = 79.7%) in overall pooled evidence and in subgroups. The current evidence is insufficient to support definitive clinical recommendations, and the observed benefit may not be generalizable across patient populations or clinical settings. To definitively clarify the therapeutic value of diaphragmatic breathing in GERD management, future randomized trials must employ standardized diagnostic criteria, uniform breathing protocols, and objective physiological outcome measures.

## Figures and Tables

**Table 1:** General characteristics of included studies.

ID	Year	Country	Study Design	Sample Size	Gender (M%)	Age	GERD Definition Criteria	Disease Subtype
Tahir et al. [26]	2024	Pakistan	Randomized controlled trial	28	64.29%	35 ± 5	Reflux Disease Questionnaire (RDQ); Gastrointestinal Symptom Rating Scale (GSRS)	Post-operative GERD NR for erosive vs. non-erosive subtype
Sadiq et al. [27]	2025	Pakistan	Randomized controlled trial (single-blinded)	22	31.82%	35 ± 10.39	GERD Health-Related Quality-of-Life Index (QoLI); Clinical diagnosis; On-demand proton pump inhibitor (PPI) usage	Classic symptomatic GERD
Suryavanshi et al. [28]	2025	India	Randomized controlled trial (single-blinded)	88	57.95%	41.4 ± 11	GERD Questionnaire (19-item severity and frequency scale)	GERD contributing to NCCP
Widjanantie et al. [29]	2023	Indonesia	Randomized controlled trial (single-blinded)	50	50.00%	36.9 ± 9.95	GERD Questionnaire (GERDQ)	Post-COVID GERD
Halland et al. [30]	2021	USA	Randomized controlled crossover/parallel trial with sham control (single-blinded outcome assessment)	33	33.33%	NR	24 h pH monitoring or pH-impedance monitoring; Upright acid exposure >5.5%	Predominantly upright GERD (mostly non-erosive)
Niu et al. [31]	2024	China	Randomized controlled trial (single-center, prospective)	60	56.67%	49 ± 15.5	Multichannel intraluminal impedance–pH monitoring (MII-pH); Clinical response to anti-reflux therapy; GERDQ	GERD with extra-esophageal manifestation (GERC)
Eherer et al. [32]	2012	Austria	Randomized controlled trial (prospective, open label)	19	52.63%	NR	Pathological pH monitoring (% time pH < 4 > 4.5%)	Classic symptomatic GERD
Sun et al. [25]	2015	China	Randomized pilot randomized controlled trial (open label)	40	42.50%	NR	Typical clinical symptoms; Endoscopy (Los Angeles classification); PPI responsiveness	Classic symptomatic GERD
Hosseini et al. [33]	2022	Iran	Randomized controlled trial (parallel design)	96	46.88%	40.38 ± 7.94	Clinical diagnosis plus Reflux Disease Questionnaire (RDQ) without endoscopy or pH monitoring	Classic symptomatic GERD
Ahmadi et al. [34]	2020	Iran	Randomized controlled trial	40	50.00%	41.4 ± 10	Clinical severity assessment (Kahrilas criteria); Typical reflux symptoms without pH monitoring or endoscopy	Classic symptomatic GERD
			Total	476	53.13%	39.9 ± 11.3		

**Table 2:** Intervention group characteristics across included studies.

ID	Sample Size	Mean Age (Years)	Type	Session Duration (min)	Sessions (Frequency)	Total Length (Weeks)	Supervision
Tahir et al. [26]	13	35 ± 5	Diaphragmatic breathing (manual costal pressure, supine)	NR	2 sessions/week (10 repetitions/session)	2	Supervised
Sadiq et al. [27]	11	NR	Diaphragmatic breathing (supine; nasal inhale–pursed lip exhale)	~5	5 sessions/day	4	Supervised initially + home program
Suryavanshi et al. [28]	42	NR	Diaphragmatic breathing + Jacobson relaxation + DNS	10–15 (relaxation component)	DB: 3×/day; Relaxation: 3×/week	4	Home-based with weekly supervision
Widjanantie et al. [29]	25	37.6 ± 9.66	Inspiratory muscle training + diaphragmatic breathing	NR	5 sessions/week	4	Supervised
Halland et al. [30]	11	58 ± 12	Slow diaphragmatic breathing (post-meal protocol)	10–30	Acute sessions during testing	Acute intervention	Supervised
Niu et al. [31]	30	45.9 ± 13.2	Supine abdominal breathing (paced breathing 6–8/min)	20	2 sessions/day	8	Initial supervision + home video monitoring
Eherer et al. [32]	10	48 ± 4	Abdominal diaphragmatic breathing (multi-position)	≥30 daily (after 1 h training)	Daily	4	Supervised + home program
Sun et al. [25]	20	48.90 ± 2.06	Diaphragm biofeedback training (DBT)	30 (hospital) + 20 (home)	Hospital: 1×/week; Home: 2×/day	8 (+ follow-up continuation)	Supervised + home program
Hosseini et al. [33]	47	40.38 ± 7.94	Diaphragmatic breathing (graded positions)	5	3 sessions/day	4	Supervised initially + home practice
Ahmadi et al. [34]	20	44.80 ± 6.59	Diaphragmatic breathing (supine hand-guided)	NR (~75 breaths/session)	5 sessions/day, 5 days/week	8	Supervised initially + home program
Total	229	44.8 ± 10.8		20.36 min per session		5.11 weeks	

**Table 3:** Comparator characteristics across included studies.

ID	Sample Size	Mean Age (Years)	Comparator Type	Comparator Details
Tahir et al. [26]	13	35 ± 5	Breathing	Supine/sitting position; deep nasal inhalation with mouth exhalation; 10 repetitions/session; 2 sessions/week; total duration 2 weeks; supervised
Sadiq et al. [27]	11	NR	Breathing	Thoracic breathing (sham)
Suryavanshi et al. [28]	40	NR	Medication	Medical therapy plus patient education only
Widjanantie et al. [29]	25	36.2 ± 10.23	Breathing	Standard diaphragmatic training only
Halland et al. [30]	10	44.0 ± 10.0	Breathing	Listening to music or observation
Niu et al. [31]	30	50.4 ± 15.4	Medication	Standard anti-reflux medication alone
Eherer et al. [32]	9	55.0 ± 4.0	Medication	On-demand PPI allowed in both groups
Sun et al. [25]	20	50.55 ± 2.28	Medication	On-demand PPI allowed in both groups
Hosseini et al. [33]	49	40.75 ± 10.04	Medication	Routine medical treatment plus dietary and lifestyle advice only
Ahmadi et al. [34]	20	20.0 ± 9.7	Medication	Omeprazole 20 mg once daily
Total	227	42.4 ± 11.6		

## Data Availability

All reported data are available on reasonable requests from the corresponding author.

## Supplementary Materials

The following supporting information can be downloaded at: https://www.mdpi.com/article/10.3390/jcm15093406/s1, PROSPERO registration report; PRISMA 2020 Checklist. References [19,20] are cited in the Supplementary Materials.
