# Supplementary material for: Efficacy and Safety of Diaphragmatic Breathing Exercises for Gastroesophageal Reflux Disease: A Systematic Review and Meta-Analysis
Source: J Clin Med. 2026 Apr 29;15(9):3406. doi: 10.3390/jcm15093406 (PMC13164494; doi:10.3390/jcm15093406)
Supplement: Supplementary file 1 [file jcm-15-03406-s001.zip › jcm-4265594-supplementary.pdf]

NIHR | National Institute for Health and Care Research

PROSPERO
International prospective register of systematic reviews

Efficacy Of Abdominal Breathing in Treatment of Gastroesophageal Reflux Disease: A Meta-analysis

Omar Abureesh, Sherif Abotaga Andrawes

Citation
Omar Abureesh, Sherif Abotaga Andrawes. Efficacy Of Abdominal Breathing in Treatment of Gastroesophageal Reflux Disease: A Meta-analysis. PROSPERO 2026 CRD420261299564. Available from https://www.crd.york.ac.uk/PROSPERO/view/CRD420261299564.

REVIEW TITLE AND BASIC DETAILS

Review title

Efficacy Of Abdominal Breathing in Treatment of Gastroesophageal Reflux Disease: A Meta-analysis

Condition or domain being studied

Abdominal Exercises; Gastroesophageal Reflux Disease; Esophagitis; Acid Reflux

Rationale for the review

This study aim to investigate the efficacy and safety of abdominal breathing exercise in reducing the symptoms severity of the Gerd and acid reflux

Review objectives

To evaluate the efficacy and safety of abdominal (diaphragmatic) breathing exercises in reducing gastroesophageal reflux disease (GERD) symptoms and related outcomes in adults, compared with usual care, sham interventions, or no intervention.

Keywords

Diaphragmatic breathing; Non-pharmacological therapy; Diaphragmatic breathing exercises

Country

United States of America

ELIGIBILITY CRITERIA

Population

Included

Adults (≥18 years) diagnosed with gastroesophageal reflux disease (GERD), including non-erosive reflux disease , confirmed clinically, endoscopically, or by validated diagnostic tools (e.g., symptom questionnaires, pH monitoring, or manometry).

Both male and female participants will be included.

Studies enrolling patients with GERD-related symptoms such as heartburn, regurgitation, belching, or reflux-associated sleep or quality-of-life impairment will be eligible.

Excluded
No restrictions will be applied based on ethnicity, geographic location, or care setting (outpatient or inpatient).

Intervention(s) or exposure(s)

Included
Breathing Exercises; Abdominal Exercises; Proton pump inhibitor

Comparator(s) or control(s)

Included
PICO tags selected: H2-receptor Antagonists; Proton pump inhibitor; Placebo

Study design

Both randomized and nonrandomized study types will be included.

Context

Studies conducted in any healthcare or community setting (including outpatient clinics, hospitals, rehabilitation centers, or home-based programs) will be eligible. No geographic restrictions will be applied. Interventions may be delivered individually or in groups provided a structured abdominal or diaphragmatic breathing protocol is described. Studies involving breathing exercises as a standalone intervention or as an adjunct to standard medical therapy will be included. Participants must have a primary diagnosis of gastroesophageal reflux disease; studies focusing exclusively on post-surgical reflux, pediatric populations, pregnancy-related reflux, or reflux secondary to major structural abnormalities (e.g., large hiatal hernia >2–3 cm or prior esophageal/gastric surgery) will be excluded.

TIMELINE OF THE REVIEW

Date of first submission to PROSPERO

09 February 2026

Review timeline

Start date: 9 February 2026. End date: 2 May 2026.

Date of registration in PROSPERO

23 March 2026

AVAILABILITY OF FULL PROTOCOL

Availability of full protocol

A full protocol has not been written.

SEARCHING AND SCREENING

Search for unpublished studies
Only published studies will be sought.

Main bibliographic databases that will be searched
The main databases to be searched are *CENTRAL - Cochrane Central Register of Controlled Trials*, *Embase.com*, *MEDLINE*, *PubMed* and *Scopus*.

Search language restrictions
The review will only include studies published in English.

Search date restrictions
There are no search date restrictions.

Other methods of identifying studies
Other studies will be identified by: *contacting authors or experts*, *looking through all the articles that cite the papers included in the review ("snowballing" or forward citation searching)*, *reference list checking (backward citation searching)* and *searching trial or study registers*.

Link to search strategy
A full search strategy is available in the full protocol as described in the *Availability of full protocol* section

Selection process
Studies will be screened independently by at least two people (or person/machine combination) with a process to resolve differences.

DATA COLLECTION PROCESS

Data extraction from published articles and reports
Data will not be extracted from publications or reports

Study risk of bias or quality assessment
Risk of bias will be assessed using: *Newcastle-Ottawa*

Data will be assessed independently by at least two people (or person/machine combination) with a process to resolve differences.

Additional information will be sought from study investigators if required information is unclear or unavailable in the study publications/reports.

Reporting bias assessment
Risk of bias due to missing results will not be assessed

Certainty assessment
Certainty of findings will not be assessed

OUTCOMES TO BE ANALYSED

Main outcomes

Primary outcomes will include changes in gastroesophageal reflux disease symptom severity and frequency.

Additional outcomes

Secondary outcomes will include GERD-related quality of life, lower esophageal sphincter pressure or esophagogastric junction metrics assessed by manometry, number of reflux episodes or esophageal acid exposure, antacid or proton pump inhibitor consumption, sleep quality, and reported adverse events related to breathing interventions.

PLANNED DATA SYNTHESIS

Strategy for data synthesis

Where studies are sufficiently homogeneous in participants, interventions, comparators, and outcomes, quantitative synthesis (meta-analysis) will be performed.

Continuous outcomes will be pooled using mean difference (MD) when the same scale is used, or standardized mean difference (SMD) when different scales are used, each with 95% confidence intervals.

Dichotomous outcomes will be pooled using risk ratios (RR) with 95% confidence intervals.

A random-effects model will be used as the primary approach due to expected clinical and methodological heterogeneity; fixed-effect models will be explored in sensitivity analyses.

Statistical heterogeneity will be assessed using the I^2 statistic and Chi-square test, and potential sources of heterogeneity will be explored via subgroup and sensitivity analyses where data allow.

If meta-analysis is not appropriate, findings will be synthesized narratively with structured tables.

CURRENT REVIEW STAGE

Stage of the review at this submission

Review stage	Started	Completed
Pilot work		
Formal searching/study identification		
Screening search results against inclusion criteria		
Data extraction or receipt of IPD		
Risk of bias/quality assessment		
Data synthesis		

Review status

The review is currently planned or ongoing.

Publication of review results

Results of the review will be published.

REVIEW AFFILIATION, FUNDING AND PEER REVIEW

Review team members

Dr Omar Abureesh (review guarantor and contact) ORCID: 0000-0002-3158-4120. Northwell Health. United States of America.

No conflict of interest declared.

Sherif Abotaga Andrawes. Northwell Health. United States of America.

No conflict of interest declared.

Named contact

Dr Omar Abureesh (oabureesh@northwell.edu). ORCID: 0000-0002-3158-4120. Northwell Health. United States of America.

Review affiliation

Northwell Staten Island

Funding source

Review has no funding and no agreed support from an academic institution and is done in authors' own time.

Peer review

There has been no peer review of this planned review.

ADDITIONAL INFORMATION

Review conflict of interest

Declared individual interests are recorded under team member details.. No additional interests are recorded for this review.

Medical Subject Headings

Adult; Breathing Exercises; Gastroesophageal Reflux; Humans

SIMILAR REVIEWS

Check for similar records already in PROSPERO

PROSPERO identified a number of existing PROSPERO records that were similar to this one (last check made on 9 February 2026). These are shown below along with the reasons given by that the review team for the reviews being different and/or proceeding.

- Breathing training as a complementary treatment of gastroesophageal reflux disease (GERD): a meta analysis and systematic review. [published 5 July 2020] [CRD42020179287]. The review was judged **not to be similar**
- Efficacy and safety of rikkunshito for gastroesophageal reflux disease: a systematic review and meta-analysis [published 9 April 2019] [CRD42019125181]. The review was judged **not to be similar**
- Meta-Analysis on the Efficacy and Safety of Acupuncture for Gastroesophageal Reflux Disease [published 31 July 2025] [CRD420251115986]. The review was judged **not to be**

similar

PROSPERO version history

- Version 1.0, published 23 Mar 2026

Disclaimer

The content of this record displays the information provided by the review team. PROSPERO does not peer review registration records or endorse their content.

PROSPERO accepts and posts the information provided in good faith; responsibility for record content rests with the review team. The guarantor for this record has affirmed that the information provided is truthful and that they understand that deliberate provision of inaccurate information may be construed as scientific misconduct.

PROSPERO does not accept any liability for the content provided in this record or for its use. Readers use the information provided in this record at their own risk.

Any enquiries about the record should be referred to the named review contact
